# Automatic Feature Segmentation in Dental Periapical Radiographs

**DOI:** 10.3390/diagnostics12123081

**Published:** 2022-12-07

**Authors:** Tugba Ari, Hande Sağlam, Hasan Öksüzoğlu, Orhan Kazan, İbrahim Şevki Bayrakdar, Suayip Burak Duman, Özer Çelik, Rohan Jagtap, Karolina Futyma-Gąbka, Ingrid Różyło-Kalinowska, Kaan Orhan

**Affiliations:** 1 Department of Oral and Maxillofacial Radiology, Faculty of Dentistry, Eskisehir Osmangazi University, 26040 Eskişehir, Turkey; 2 Health Services Vocational School, Gazi University, 06560 Ankara, Turkey; 3 Eskisehir Osmangazi University Center of Research and Application for Computer-Aided Diagnosis and Treatment in Health, 26040 Eskişehir, Turkey; 4 Division of Oral and Maxillofacial Radiology, Department of Care Planning and Restorative Sciences, University of Mississippi Medical Center School of Dentistry, Jackson, MS 39216, USA; 5 Department of Oral and Maxillofacial Radiology, Faculty of Dentistry, Inonu University, 44000 Malatya, Turkey; 6 Department of Mathematics-Computer, Faculty of Science, Eskisehir Osmangazi University, 26040 Eskisehir, Turkey; 7 Department of Dental and Maxillofacial Radiodiagnostics, Medical University of Lublin, 20-059 Lublin, Poland; 8 Department of Oral and Maxillofacial Radiology, Faculty of Dentistry, Ankara University, 0600 Ankara, Turkey; 9 Ankara University Medical Design Application and Research Center (MEDITAM), 0600 Ankara, Turkey

**Keywords:** artificial intelligence, periapical radiographs, deep learning, oral diseases, oral findings

## Abstract

While a large number of archived digital images make it easy for radiology to provide data for Artificial Intelligence (AI) evaluation; AI algorithms are more and more applied in detecting diseases. The aim of the study is to perform a diagnostic evaluation on periapical radiographs with an AI model based on Convoluted Neural Networks (CNNs). The dataset includes 1169 adult periapical radiographs, which were labelled in CranioCatch annotation software. Deep learning was performed using the U-Net model implemented with the PyTorch library. The AI models based on deep learning models improved the success rate of carious lesion, crown, dental pulp, dental filling, periapical lesion, and root canal filling segmentation in periapical images. Sensitivity, precision and F1 scores for carious lesion were 0.82, 0.82, and 0.82, respectively; sensitivity, precision and F1 score for crown were 1, 1, and 1, respectively; sensitivity, precision and F1 score for dental pulp, were 0.97, 0.87 and 0.92, respectively; sensitivity, precision and F1 score for filling were 0.95, 0.95, and 0.95, respectively; sensitivity, precision and F1 score for the periapical lesion were 0.92, 0.85, and 0.88, respectively; sensitivity, precision and F1 score for root canal filling, were found to be 1, 0.96, and 0.98, respectively. The success of AI algorithms in evaluating periapical radiographs is encouraging and promising for their use in routine clinical processes as a clinical decision support system.

## 1. Introduction

Although clinical and visual evaluation in dentistry is the first step in diagnosis, radiographic examination is often used as an auxiliary method in the main diagnosis [1]. Dentists often use periapical radiography, bitewing radiographs and panoramic radiographs in the clinic. Panoramic radiographs are routinely preferred because they can display all dentoalveolar structures together, but this technique is not as diagnostic as periapical radiographs due to the enlargement and geometric distortions that occur in the images [2]. Periapical radiographs are the most preferred intraoral radiography technique as an aid to dental carious lesion detection, examination of restorations, inter radicular radiolucency, root canal morphology, evaluation of alveolar bone level, periodontal ligament space, and endodontic treatments. While the experience and knowledge of the physician play an important role in the interpretation of these radiographs, factors such as radiographic technique errors, contrast, and magnification may cause misinterpretation of the image [3]. For this reason, it is very meaningful for dentists to develop an automatic detection method that assists in diagnosis and treatment evaluation stages on periapical radiographs.

Artificial intelligence (AI) is described as the capacity to simulate human intelligence, and today, an AI system has been developed and is being widely used in different fields, including medicine and dentistry [4]. AI algorithms provide benefits in detecting diseases while avoiding unnecessary procedures [5]. In the field of radiology, training AI systems became easy through data provided by a large number of archived digital images [6]. Machine learning (ML) is a subset of AI that uses training datasets to create algorithms that can learn on its own, providing computers or systems with the ability to learn and improve performance automatically without human intervention [7]. The main goal in machine learning is to create mathematical models that can be trained to produce desired results when fed with input data. Machine learning has been developed for many years and has many subclasses. The most popular of these subgroups today is deep learning (DL) algorithms. Simple network architectures with numerous layers are referred to as “shallow” learning neural networks, which were one of the first algorithms to be devised. DL neural networks are network designs that make use of numerous massive layer networks. Convolutional neural networks (CNNs) are typically employed to analyze massive and complicated images [8]. Neural networks are mathematical models that mimic the human brain [9]. A general CNN architecture includes several convolutional layers that can break down an image into smaller pieces that allow easy processing. These layers’ outputs are routed into the pooling layer. In the pooling layer, the data size and existing noise is reduced. These two layers are fed into the neural network that generates a probability map from images containing a desired target for detection [10]. In medical imaging, CNNs are the most popular deep learning architectures and by utilizing original images CNNs can automatically create visual features [11]. In the field of dentistry, there is an increasing number of studies that use deep learning for diagnosis, screening, and decision making. Most of the studies are aimed at evaluating the diagnostic performance of deep learning models developed for segmentation, classification, or anatomic landmark detection. Numerous studies demonstrated that diagnosis using artificial intelligence models, such as CNN architectures, is very promising especially in carious lesion detection [9,12,13,14,15], periodontal disease detection [16,17], cephalometric analysis [18], periapical lesion detection [19], detection of atherosclerotic carotid plaques [20], detection of taurodont teeth [21], and segmentation and classification of Sella turcica [22] findings.

The purpose of this study is to evaluate automatic segmentation performance of features in periapical radiographs by DL based AI model developed using U-Net algorithm. 

## 2. Materials and Methods

### 2.1. Patient Selection

Periapical radiographs used for various diagnostic purposes were obtained from the archive of Eskisehir Osmangazi University, Faculty of Dentistry. The dataset includes 1169 periapical radiographs taken from adults between January 2016 and June 2020. Radiographic images with poor image quality and artifacts that may affect the diagnosis were excluded from the study. The research protocol was approved by the Eskisehir Osmangazi University Non-Interventional Clinical Research Ethics Committee and follows the principles of the Declaration of Helsinki (decision date and number: 15 June 2021, 44).

### 2.2. Radiographic Dataset

All periapical radiographs were taken with the ProX periapical X-ray unit (Planmeca, Helsinki, Finland) with the following parameters: 220–240-V, 60 kVp, 2 mA, and 0.05 s scan time using ProScanner Phosphor Plate and Scanning System (Planmeca, Helsinki, Finland).

### 2.3. Image Evaluation

As a research assistant with 2 years of experience and a dento-maxillofacial radiologist with 12 years of experience performed labeling on periapical radiographs for different findings using CranioCatch annotation software (CranioCatch, Eskişehir, Turkey).

### 2.4. Deep Convolutional Neural Network

**Figure 1:**
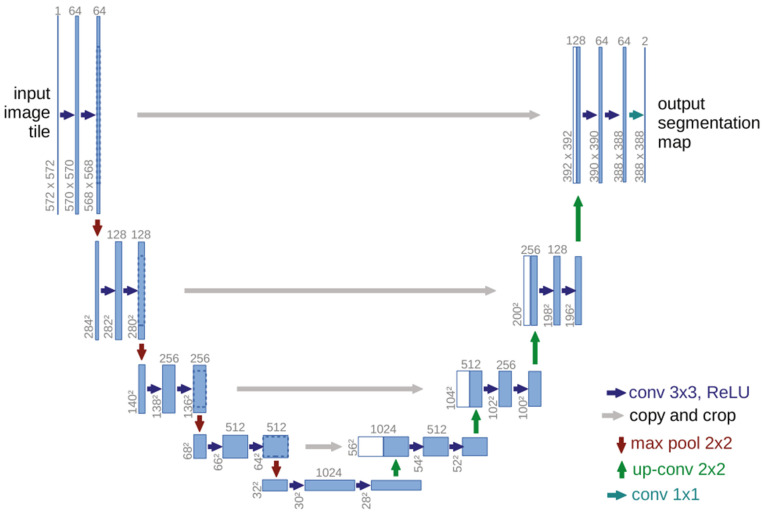
Overview of U-Net Architecture.

DL was performed using the U-Net model implemented with the PyTorch library (version 1.4.0). Blue boxes in Figure 1’s U-Net design represent multi-channel feature maps and white boxes reproduce feature maps. The numbers above the blue boxes correspond to the channel numbers. This architecture consists of narrowing and widening roads. The widening path is roughly symmetrical with the narrowing path, forming a U-shaped structure. The narrowing side (left) of the U shape is an encoder section where feature extraction is performed. Each block in this section consists of two 3 × 3 convolution and 2 × 2 max pooling layers with rectified linear unit (ReLu) activation. The number of feature channels are doubled at each subsampling step. On the expanding side (right) of the U-shape, the decoding step and sampling operation is performed where the amount of feature channels is halved via a 2 × 2 deconvolution. The feature map of each transmitted convolution layer is combined with a feature map of a resolution similar to that of the encoder section. In the final stage, 1 × 1 convolution is applied to reduce the feature map to the required number of channels and provide segmentation. (Figure 1) [23]. Due to sequential convolutional layers, this architecture allows more accurate segmentation and it can successfully segment images with limited training data. For these reasons, it is generally preferred in image segmentation in the medical field. 

### 2.5. Model Pipeline and Training Phase

**Figure 2:**
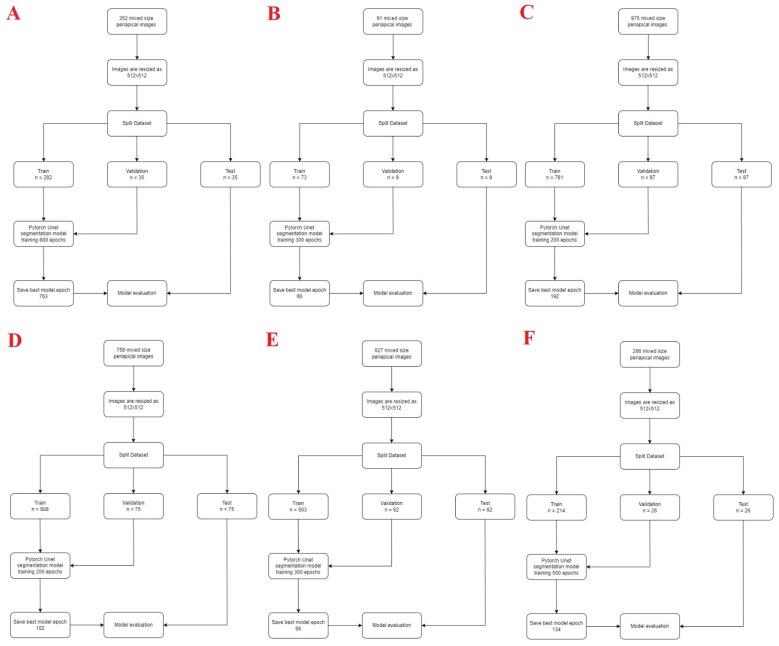
Diagram of the dental conditions’ segmentation model development steps ((**A**) carious lesion, (**B**) crown, (**C**) dental pulp, (**D**) filling, (**E**) root canal filling, (**F**) periapical lesion).

The PyTorch library was used in this study. It is an open-source library that aims to remove this barrier for both researchers and practitioners. Python open-source programming language (v.3.6.1; Python Software Foundation, Wilmington, DE, USA) and PyTorch library were used for model development. The training method was applied using computer equipment of Eskisehir Osmangazi University Faculty of Dentistry Dental-AI Laboratory including Dell PowerEdge T640 Calculation Server (Dell Inc., Austin, TX, USA), Dell PowerEdge T640 GPU Calculation Server (Dell Inc., TX, USA), and Dell PowerEdge R540 Storage Server (Dell Inc., TX, USA). Anonymized mixed-size periapical images were resized to 512 × 512 to increase image visual quality. This study applied image enhancement techniques such as intensity normalization and Contrast Limited Adaptive Histogram Equalization (CLAHE). Approximately 80% of the data set are divided into 3 parts: 80% training, 10% testing, and 10% validation. The AI model was trained using different epoch values for each situation. The model’s learning rate was found to be 0.0001. The testing groups data were not reused (Figure 2).

***Statistical Analysis***

A confusion matrix was used to evaluate model performance. This matrix was a meaningful table that summarizes the predicted situation with the actual situation.

***Metrics Calculation Procedure***

The following metrics were used to assess the model’s success:

- *True positive (TP):* dental diagnoses correctly detected and segmented.
- *False positive (FP):* dental diagnoses detected but incorrectly segmented.
- *False negative (FN):* dental diagnoses incorrectly detected and segmented.

The performance metrics of the model were determined according to the formulas using the following TP, FP, and FN numbers.

- **Sensitivity, true positive rate (TPR):** TP/(TP + FN)
- **Precision, positive predictive value (PPV):** TP/(TP + FP)
- **F1 score:** 2TP/(2TP + FP + FN)

***Intersection over Union (IoU):*** Intersection over Union (IoU) is a typical assessment approach that uses true positives, false positives, and false negatives in Pascal VOC 2012. The IoU metric displays area where the outcome of the suggested approach and the precise reference space overlap (ground truth) [24]. In this study, this area was accepted as a TP (true positive) if it was greater than 50% and FP (false positive) if it was smaller.

## 3. Results

**Figure 3:**
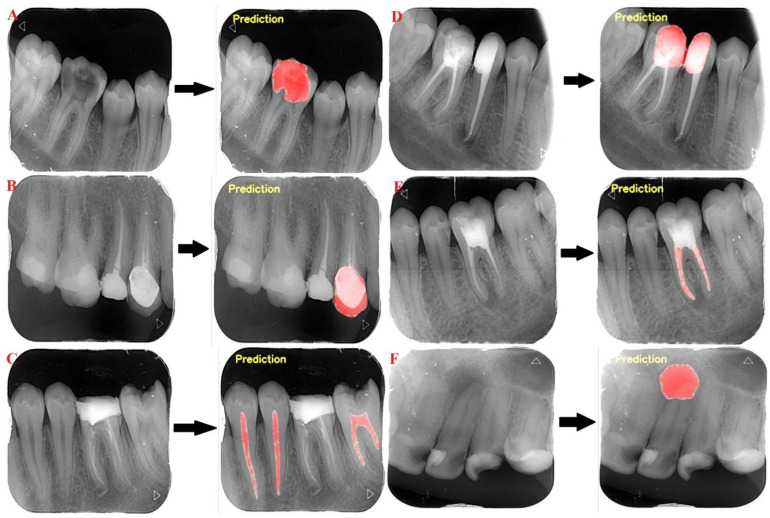
Dental conditions’ segmentation on periapical radiographs using the AI model ((**A**) caries, (**B**) crown, (**C**) dental pulp, (**D**) filling, (**E**) root canal filling, (**F**) periapical lesion).

The AI models based on deep-learning models improved the success rate of carious lesion, crown, dental pulp, dental filling, periapical lesion, and root canal filling segmentation in periapical images. All test images were correctly segmented by the developed AI model (Figure 3).

Sensitivity, precision, and F1 score for carious lesion, crown, dental pulp, filling, periapical lesion, root canal filling were found to be 0.82, 0.82, and 0.82, respectively; 1, 1, and 1, respectively; 0.97, 0.87, and 0.92, respectively; 0.95, 0.95, and 0.95, respectively; 0.92, 0.85, and 0.88, respectively; and 1, 0.96, and 0.98, respectively (Table 1 and Table 2).

## 4. Discussion

The development of deep learning and neural network methods has accelerated the use of AI in medicine and dentistry [19]. Many studies are using AI in the field of dentistry. Hamdan et al. [25], as a result of their studies investigating the contribution of deep learning tools to the evaluation of radiolucent areas in the apical regions of the teeth, concluded that the diagnostic efficiency of clinicians in determining apical radiolucency on periapical radiographs increased with the AI model. Chen et al. [26] conducted a study to detect and number teeth faster in dental periapical films with Faster R-CNN in the TensorFlow library. Their studies have shown that predictions and recalls are 90% accurate using Faster R-CNN. The responses of three dentists who examined the independent dataset were compared with the responses of the system, and they found success in the study close to that of dentists new to the profession [26]. The objective of Görürgöz et. al. [27] was to assess how well the Faster Region-Based Convolutional Neural Network (R-CNN) algorithm performed when it came to identifying and numbering teeth on periapical radiographs. In their study, evaluating 1686 periapical radiographs, a pre-trained model (GoogLeNet Inception v3 CNN) was used for preprocessing and transfer learning techniques were applied for dataset training. An AI algorithm based on R-CNN initial architecture has been designed to automatic detect and numbering teeth. Of the 864 teeth in the 156 periapical radiographs, 668 were correctly numbered in the test dataset. The F1 score, precision, and sensitivity were specified as 0.8720, 0.7812, and 0.9867, respectively [27]. To assess the efficacy of deep CNN algorithms for the identification and diagnosis of dental caries on periapical radiographs, Lee et al. [12] used the GoogLeNet Inception v3 CNN network for preprocessing and transfer learning on 3000 periapical radiographs. The diagnostic accuracy of the models was found to be 89% in premolar models, 88% in molar models, and 82% in premolar-molar models, and the researchers stated that the deep CNN algorithm provides a very good success in detecting dental caries in periapical radiographs [12]. In another study on periapical and bitewing radiographs, composite, amalgam, and metal-ceramic restorations were segmented with a ResNet34 architecture-based CNN model, and it was reported that the model, with AUC values of 0.95, 0.95, and 1.00, respectively, showed high diagnostic performance [28]. 

Khan et al. [3] labeled carious lesion, alveolar bone loss, and inter radicular radiolucency on periapical radiographs using deep learning techniques. They claimed that the U net+ Densenet 121 design had the greatest performance in the validation dataset when they examined the results of deep learning architectures to select the most appropriate architecture for automated analysis [3]. Lee et al. [16], in their study on periapical radiographs, wanted to develop a computer-aided detection system based on the CNN algorithm and evaluate the potential utility and accuracy of this system for the diagnosis and prediction of periodontal disease in teeth. The overall diagnostic accuracy was 81.0% for premolar teeth, the highest diagnostic accuracy (82.8%) for severe periodontal disease, and the lowest (77.3%) for moderate periodontal disease. The overall diagnostic accuracy was 76.7% for molar teeth, the highest (81.3%) for severe periodontal disease, and the lowest (70.3%) for moderate periodontal disease. They claimed that the deep CNN algorithm can help with periodontal disease detection and prediction [16]. Images of 801 patients were used in a study to evaluate the classification of four different implant types on periapical radiographs of deep neural networks. Images containing Brånemark Mk TiUnite, Dentium Implantium, Straumann Bone Level, and Straumann Tissue Level implant types were used, and SqueezeNet, GoogLeNet, ResNet-18, MobileNet-v2, and ResNet-50 were evaluated in order to select the best pre-trained network design. All five models were reported to have test accuracy of over 90%. According to the findings of this work, even with a small mesh size and few images, a CNN can assess implant photos and automatically classify four specific implant fixture types with high accuracy [29]. In another study, in which a total of 1800 digital periapical radiographic images were evaluated with the software developed through CNN to detect and identify three different dental implant brands, accuracy values were found to be 99.78% for group training data, 99.36% for group test data, and 85.29% for validation data [30]. Cha et al. [31] aimed to measure bone loss around the implant and evaluate its severity in 708 periapical radiographs. They used transfer learning to train a modified region-based CNN (R-CNN) using data from the Microsoft Common Objects dataset. In this study, in which radiographic bone loss was measured and classified by identifying key points, it was stated that there was no statistically significant difference between the modified R-CNN model and the evaluation of the dentist in determining the landmarks around dental implants [31]. Li et al. [32] made research to evaluate success of automatic detection dental caries and periapical periodontitis on periapical radiographs using two cascaded ResNet-18 backbones and two individual convolutional layers. The deep learning model automatically recognized caries with an F1-score of 0.829 and periapical periodontitis with an F1-score of 0.828. The AI model showed remarkably higher success than young dentists. They concluded that the AI could develop the accuracy and consistency of assess tooth caries and periapical periodontitis on periapical radiographs based on these study results. [32] Another study conducted by Chen et al. [33] purposed develop CNN based AI model for detection of lesions on periapical radiographs and assess the performances in terms of disease types, severe of lesion, and train methods. Generally, precision and recall values were found as between 0.5 and 0.6 on different type of disease for lesions detection. The effect of train methods, disease type, and severe of lesions had statistically significance on performances (P<.001). The study emphasized that deep CNNs has capable to detect diseases on periapical radiographs. [33] Duong et al. [34] presented an automate recognize carious lesions algorithm on tooth occlusal surfaces in smartphone images according to International Caries Detection and Assessment System using a group of extracted teeth. The achieved images were assessed and annotated into three different classes: “No Surface Change (NSC)”; “Visually Non-Cavitated (VNC)”; and “Cavitated” (C). An automated two-step SVM classification system was developed for caries detection. The accuracy, sensitivity, and specificity were found as 92.37%, 88.1%, and 96.6%, respectively, in C vs. (VNC + NSC) classification and they were found as 83.33%, 82.2%, and 66.7%, respectively, in VNC vs. NSC classification. [34] Alevizakos et al. [35] developed an AI system for training diagnosis and differentiation with molar incisor hypomineralization (MIH) and carious lesion, amelogenesis imperfecta, dental fluorosis in clinical pictures. ResNet34, ResNet50, AlexNet, VGG16 and DenseNet121 were performed on the datasets. The precision of VGG16 network was the lowest with 83.98% for amelogenesis imperfecta. Dense121 showed the highest values with 92.86%. The carious lesion detection rate was 100% in the resNet50 group. [35]

In the presented study, U-Net based AI algorithm was developed to segmentation of different dental findings in dental periapical radiographs. The sensitivity, precision, and F1 value was found as up to 80%. Although the presented study has some limitations including one type X-ray machine used to take images, didn’t use external data set, lack of observers with different experiences, didn’t use different CNN models. This developed AI model using U-Net model improved the success rate of, carious lesion, crown, dental pulp, dental filling, periapical lesion, and root canal filling segmentation on dental periapical images. Future studies should be held using more data and eliminated these limitations.

## 5. Conclusions

The result of this study demonstrates the potential of deep learning systems based on CNN architectures to assist dentists in segmenting different features in periapical images. Having sufficient training data is necessary to achieve successful results in training deep neural networks. The success rate can be increased with more data; therefore, it can be ensured that physicians use time efficiently in the diagnosis process. 

## Figures and Tables

**Table 1:** Data distribution and training parameters.

	Periapical Radiograph Numbers for Training	Label Numbers for Training	Periapical Radiograph Numbers for Test	Label Numbers for Test	Periapical Radiograph Numbers for Test	Label Numbers for Test	Learning Rate	Epoch
Carious lesion	352	577	35	59	35	53	0.0001	800
Crown	91	108	9	11	9	12	0.0001	300
Dental Pulp	975	3482	97	347	97	348	0.0001	200
Filling	758	1600	75	169	75	161	0.0001	200
Root Canal Filling	627	1389	62	138	62	165	0.0001	300
Periapical Lesion	266	327	26	34	26	30	0.0001	500

**Table 2:** Estimated performance measurement based on the AI model.

	True-Positive (TP)	False- Positive (FP)	False- Negative (FN)	Sensitivity (TP/(TP + FN))	Precision (TP/(TP + FP))	F1 Score (2TP/2TP + FP + FN))
Carious lesion	34	7	7	0.82	0.82	0.82
Crown	12	0	0	1	1	1
Dental Pulp	274	40	6	0.97	0.87	0.92
Filling	129	6	6	0.95	0.95	0.95
Root Canal Filling	110	4	0	1	0.96	0.98
Periapical Lesion	24	4	2	0.92	0.85	0.88

## Data Availability

Data is available from the corresponding author upon reasonable request.
